# Real-time spatial health surveillance: Mapping the UK COVID-19 epidemic

**DOI:** 10.1016/j.ijmedinf.2021.104400

**Published:** 2021-05

**Authors:** Richard Fry, Joe Hollinghurst, Helen R Stagg, Daniel A Thompson, Claudio Fronterre, Chris Orton, Ronan A Lyons, David V Ford, Aziz Sheikh, Peter J Diggle

**Affiliations:** a Health Data Research, UK; b Medical School, Lancaster University, UK; c Swansea University Medical School, UK; d Usher Institute, University of Edinburgh, UK

## Abstract

**Introduction** The COVID-19 pandemic has highlighted the need for robust data linkage systems and methods for identifying outbreaks of disease in near real-time. **Objectives** The primary objective of this study was to develop a real-time geospatial surveillance system to monitor the spread of COVID-19 across the UK. **Methods** Using self-reported app data and the Secure Anonymised Information Linkage (SAIL) Databank, we demonstrate the use of sophisticated spatial modelling for near-real-time prediction of COVID-19 prevalence at small-area resolution to inform strategic government policy areas. **Results** We demonstrate that using a combination of crowd-sourced app data and sophisticated geo-statistical techniques it is possible to predict hot spots of COVID-19 at fine geographic scales, nationally. We are also able to produce estimates of their precision, which is an important pre-requisite to an effective control strategy to guard against over-reaction to potentially spurious features of ’best guess’ predictions. **Conclusion** In the UK, important emerging risk-factors such as social deprivation or ethnicity vary over small distances, hence risk needs to be modelled at fine spatial resolution to avoid aggregation bias. We demonstrate that existing geospatial statistical methods originally developed for global health applications are well-suited to this task and can be used in an anonymised databank environment, thus preserving the privacy of the individuals who contribute their data.

## Highlights

• First high-resolution mapping of COVID-19 prevalence in the UK.
• Sophisticated geospatial methods to predict community prevalence.
• Longitudinal change in COVID-19 prevalence.
• Team science approach using SAIL and the HDR UK network.

## 1 Introduction

On 11th March 2020, the World Health Organization declared a pandemic of COVID-19 caused by the SARS-CoV-2 coronavirus [1]. By this date, the UK had reported 373 confirmed COVID-19 cases and six deaths [2]. Up to 15th July 2020, these figures had risen to 291,911 and 45,053 [3]. Lockdowns governing the movement of the population and opening of shops and other facilities, initially imposed across the entire country on 23rd March 2020 [4], have been a key tool in the government's response to COVID-19. Since that date, detection of local variations in infection rates has been critical for controlling the spread of SARS-CoV-2 [5], including ascertaining the level of required local public health response across the UK. A key example of this was the implementation of the first ’local lockdown’ in Leicester on 30th June 2020, in response to a cluster of COVID-19 accounting for approximately one in ten of all new disease cases across the country in the preceding week [6].

The COVID Symptom Study app (Zoe Global Limited, King's College London) was released publicly on 24th March 2020 [7], the day after the UK-wide lockdown rules were first imposed. The app collects postcode of residence at the time of registration and daily updates on self-reported COVID-19-associated symptoms. The Secure Anonymised Information Linkage (SAIL) Databank facilitates robust secure storage and use of anonymised person-based data for research to improve health, well-being and services [8], [9]. During the pandemic, SAIL has been receiving daily updates of the COVID Symptom Study app data, facilitating near real-time health surveillance of COVID-19 across the UK.

To help understand the localised spread and flare up of the disease we adapted existing statistical methodology [10] for the analysis of geo-referenced health outcome data to map, at Lower-layer Super Output Area (LSOA) resolution (Datazone in Scotland and Super Output Area in Northern Ireland), the prevalence of positive symptom reports amongst app users over a rolling 14-day period, together with associated limits of statistical uncertainty. Notwithstanding the limitations of this self-reported health outcome, these maps provide the first fine-scale, UK-wide assessment of the geographical distribution of probable COVID-19 infections, and have been used by the devolved administrations in each country for pandemic planning [11].

## 2 Methods

### 2.1 Population sampling

Across the UK, as of 1st November 2020, 4,198,408 individuals had registered on the COVID Symptom Study app. Use of the app is voluntary and thus the population sample is non-random. Users of the app had to have access to an internet-enabled telephone, although reporting for multiple individuals in the same household was instigated on 1st May 2020 for those people who could not access or use the app [12]. At the time of registration, individuals report baseline demographic and clinical information (e.g. underlying health conditions), as well as postcode of residence. Self-reported data on COVID-19-associated symptoms, including fever and persistent cough, are recorded for any day on which an individual reports. For a full metadata summary see the HDR Gateway deposit [13].

The SAIL Databank acts as a secure gateway to the ZOE app data for the whole of the UK. Data are made available daily via a secure data transfer and processed into a SQL DB2 database. Access to the data is via a secure remote desktop login following approval for a project via an application to the SAIL Information Governance Review Panel (IGRP). In light of the COVID-19 crisis, IGRP applications were typically approved within 24-hours. Prior to transfer of the data to the SAIL Databank, postcode data were aggregated to LSOA level using the Office for National Statistics (ONS) postcode lookup directory [14] to maintain an app user's privacy.

### 2.2 Informatics

Data were extracted from the SAIL Databank using SQL and processed to generate suitable inputs for the geospatial modelling. Where an individual reported their symptoms more than once in a day, the last record was taken. Likely instances of COVID-19 were calculated through either a) the presence of high fever and persistent cough (’classic symptoms’) or b) an algorithm developed by the King's College team, which used an array of symptoms and other characteristics (persistent cough, skipping meals, loss of smell, gender, age and fatigue; ’multi-symptom algorithm’), [15]. The multi-symptom algorithm captures more app users who may be displaying symptoms which are not specific to COVID-19 (e.g. fatigue, shortness of breath, diarrhoea) and may not be reporting classic symptoms [15]. As a result there are noticeable differences in the prevalence estimates shown in Fig. 2 which vary over the study period. However, when taken in combination Menni et al. [15] have demonstrated that the multi-symptom algorithm performs well in predicting COVID-19 cases. The denominator of users in each LSOA for each analysis was calculated using a 14-day retrospective window i.e. the number of individuals who had reported data to the app at any time during that period. The numbers of app users and cases were then aggregated to the LSOA level. The resulting data for each LSOA consisted of its population-weighted centroid, x, the number of people who used the app at least once over the time-period in question, n, and the number of those who were predicted to have COVID-19 at least once within the time-period, y.

**Fig. 1:**
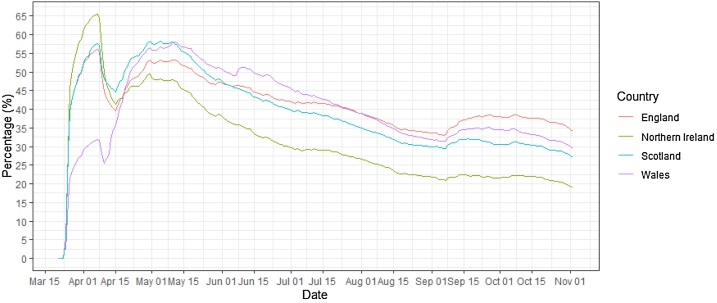
Longitudinal trajectories of app use for each of the four UK countries up to 1st November 2020, active within a fourteen-day rolling time-window.

**Fig. 2:**
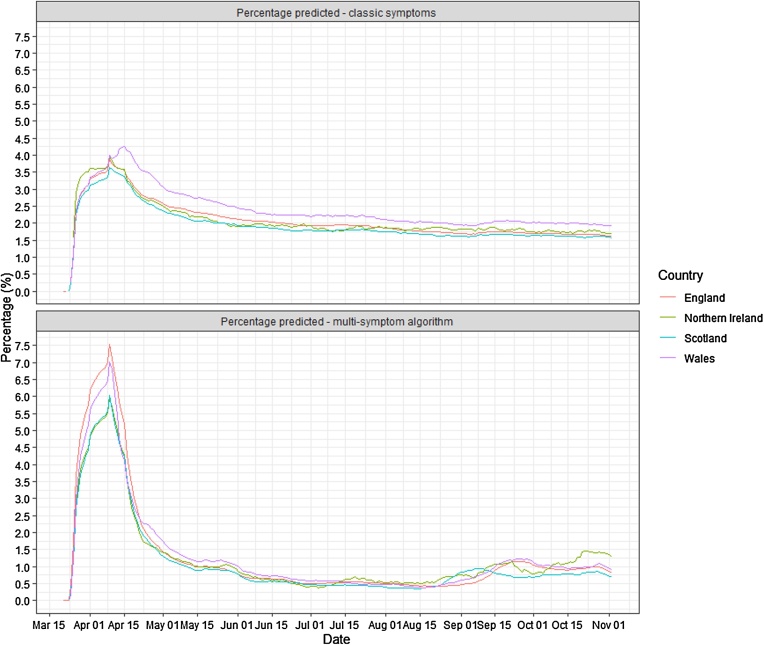
Longitudinal trajectories of symptom-prevalence (percentage of active users reporting symptoms) for each of the four UK countries up to 1st November 2020, based on classic symptoms (upper panel) and multi-symptom algorithm (lower panel).

### 2.3 Geospatial statistical model and inference

Our statistical model is a geospatial extension of the logistic regression model for binomial (numerator/denominator) data, in which the log-odds of the probability, P(x), of at least one positive symptom report is the unobserved realisation of a spatially correlated stochastic process and, conditional on P(x), the corresponding numerator y follows a binomial distribution with denominator n. The model has three parameters that determine the mean and variance of P(x) and the rate at which the correlation between the values of P(x) at two different locations decays with increasing distance between them. The standard way to present a prevalence map is as a set of point estimates. However, point estimates tend to be most extreme for locations with small denominators, because of their relatively sampling variation. Another common practice is to apply a series of tests for statistically significant local departures from the area-wide prevalence, with significantly large or small locations labelled as “hot-spots” and “cold-spots,” respectively [16], [17]. A limitation of this approach is that statistically significant local departures from the area-wide average are more likely to be associated with large denominators, even if the size of the local departure is too small to be of public health significance. We argue that a more satisfactory way to conduct prevalence mapping is as a prediction problem. The predictive distribution of the complete prevalence surface is its probability distribution conditional on all of the available data. By sampling from this distribution we can, *a fortiori*, derive a sample from whatever property of the prevalence surface is of interest. For the results presented in this paper we chose to map four summaries: the mean, a point prediction of prevalence; the 5% and 95% quantiles, which together measure the uncertainty associated with each point prediction; and the probability that the prevalence in the LSOA in question is greater than the country-wide average for each devolved nation. Arguably, If the primary aim of the mapping is hot-spot detection, additional useful summaries would be the probabilities that local prevalence exceeds each of a set of thresholds that would be considered large enough to trigger one of several possible public health responses. Patches of mapped probabilities close to 1 or 0 would then indicate both the geographical extent and magnitude of local hot-spots and cold-spots, respectively.

We estimated the parameters using Monte Carlo maximum likelihood and used the fitted model to draw samples from the joint predictive distribution of P(x) over all LSOA population weighted centroids. Parameters were re-estimated separately for each of the UK's constituent countries, in each case using data aggregated over a rolling 14-day time-period. In the Supplementary Material we describe in detail how we developed and fitted the particular model that we used for our application to the COVID-19 app data.

The complete prediction for each LSOA is a probability distribution for its underlying prevalence. This distribution can be summarised as the user wishes. We chose to map four summaries: the mean, a point prediction of prevalence; the 5% and 95% quantiles, which together measure the uncertainty associated with each point prediction; and the probability that the prevalence in the LSOA in question is greater than the country-wide average for each devolved nation, with mapped values close to 1 or 0 indicating “hot-spots” and “cold-spots” respectively. If the primary aim of the mapping is hot-spot detection, additional useful summaries would be the probabilities that local prevalence exceeds each of a set of thresholds representing increasing multiples of the country-wide average. Patches of mapped probabilities close to 1 would then indicate both the geographical extent and magnitude of local hot-spots. Predicted prevalence data were summarised for the whole of the UK, and for each of its four constituent countries.

## 3 Results

### 3.1 App users

Table 1 summarises the users of the app for the UK as of 1st November 2020. Across the UK users were predominately female, white, between 30 and 55 and lived in the least deprived areas. It is also worth noting that the 20% of users did not provide a full postcode thereby limiting the utility of these users data provision for the purposes of high-resolution spatial modelling.

**Table 1:** Summary of registered ZOE Symptom Study App users as of 1st November 2020.

	Number	Percentage
Registered app users	4198408	100.0%

Age		
Median (IQR)	40 (30,55)	
0–10	192431	4.6%
15–20	378000	9.0%
25–30	728487	17.4%
35–40	762011	18.1%
45–50	720108	17.2%
55–60	584101	13.9%
65–70	368274	8.8%
75–80	100750	2.4%
85+	23529	0.6%
Not Recorded	330717	7.9%

*Gender*		
Female	2342803	55.8%
Male	1521609	36.2%
Prefer not to say	3014	0.1%
Intersex	301	<0.1%
Not Recorded	330681	7.9%

*Ethnicity*		
Hispanic	64	0.0%
Other	13961	0.3%
Prefer not to say	12371	0.3%
Asian (UK)	64894	1.5%
Black (UK)	18176	0.4%
Chinese	10999	0.3%
Middle Eastern	11709	0.3%
Mixed (other)	36736	0.9%
Mixed (White/Black)	18974	0.5%
White (UK)	2817129	67.1%
US Residents	2163	0.1%
Not Recorded	1191232	28.4%

*Deprivation (Townsend Quintile)*		
1. Least Deprived	924910	22.0%
2	837050	19.9%
3	702876	16.7%
4	544344	13.0%
5. Most Deprived	429120	10.2%
Not Recorded	760108	18.1%

### 3.2 App usage over time

There was no national government requirement for members of the public to use the app. In each country, the number of people registering to use the system increased rapidly in the early weeks of its availability, and more slowly thereafter (Fig. 1). In England, Scotland and Wales the number of active users (people who recorded one or more app submission in the preceding 14 days) also increased between mid-April and early May, but declined thereafter, from a peak of around 60% in early May to around 45% in mid July (Fig. 1). In Northern Ireland, where the app is, in effect, competing with Northern Ireland's own app [18] the percentage of active users peaked at around 50% in early May and had declined to about 30% by mid July.

### 3.3 Predicted prevalence of COVID-19 over time

Predicted disease prevalence over time, weighted for population size within each LSOA, was plotted using both classic symptoms and the multi-symptom algorithm. Both provided similar patterns of predicted disease prevalence for the first two weeks of data collection (Fig. 2), the figures from the multi-symptom algorithm were higher than those using classic symptoms (classic symptoms 3.6% to 4.3% across the four countries at their peak, multi-symptom algorithm 6.0% to 7.5%) but diverged thereafter. Using classic symptoms, predicted prevalence slowly declined to mid-May and then was approximately stable at slightly more than 2% in Wales, slightly less than 2% in England, Scotland and Northern Ireland (Fig. 2, upper panel). Using the multi-symptom algorithm, the decline was more rapid, stabilising around two weeks earlier at approximately 0.4% in all four countries (Fig. 2, lower panel).

### 3.4 Predictive mapping

**Fig. 3:**
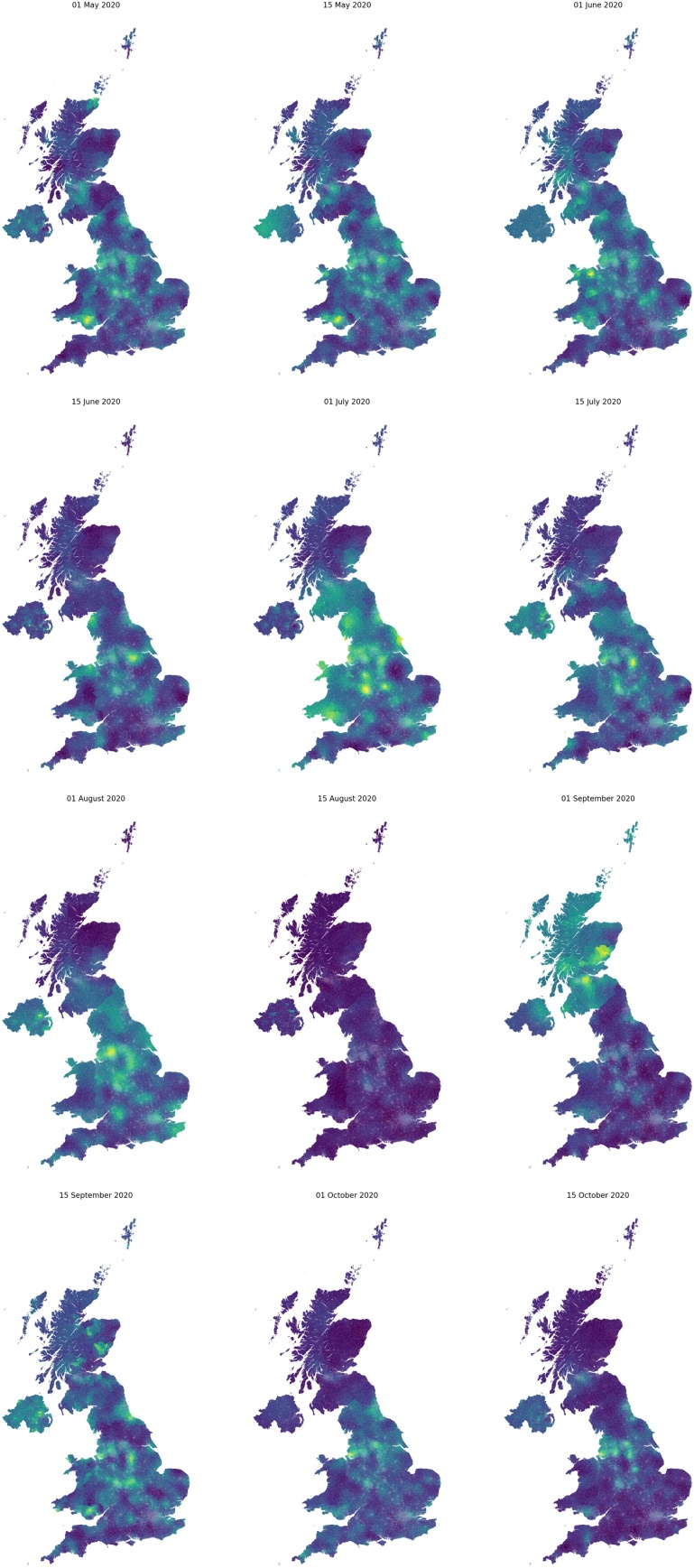
LSOA Level COVID-19 prevalence predictions using a 14-day window 1st May – 1st November 2020. Purples represent low values, green mid range and yellow high values. (For interpretation of the references to color in this figure legend, the reader is referred to the web version of this article.)

**Fig. 4:**
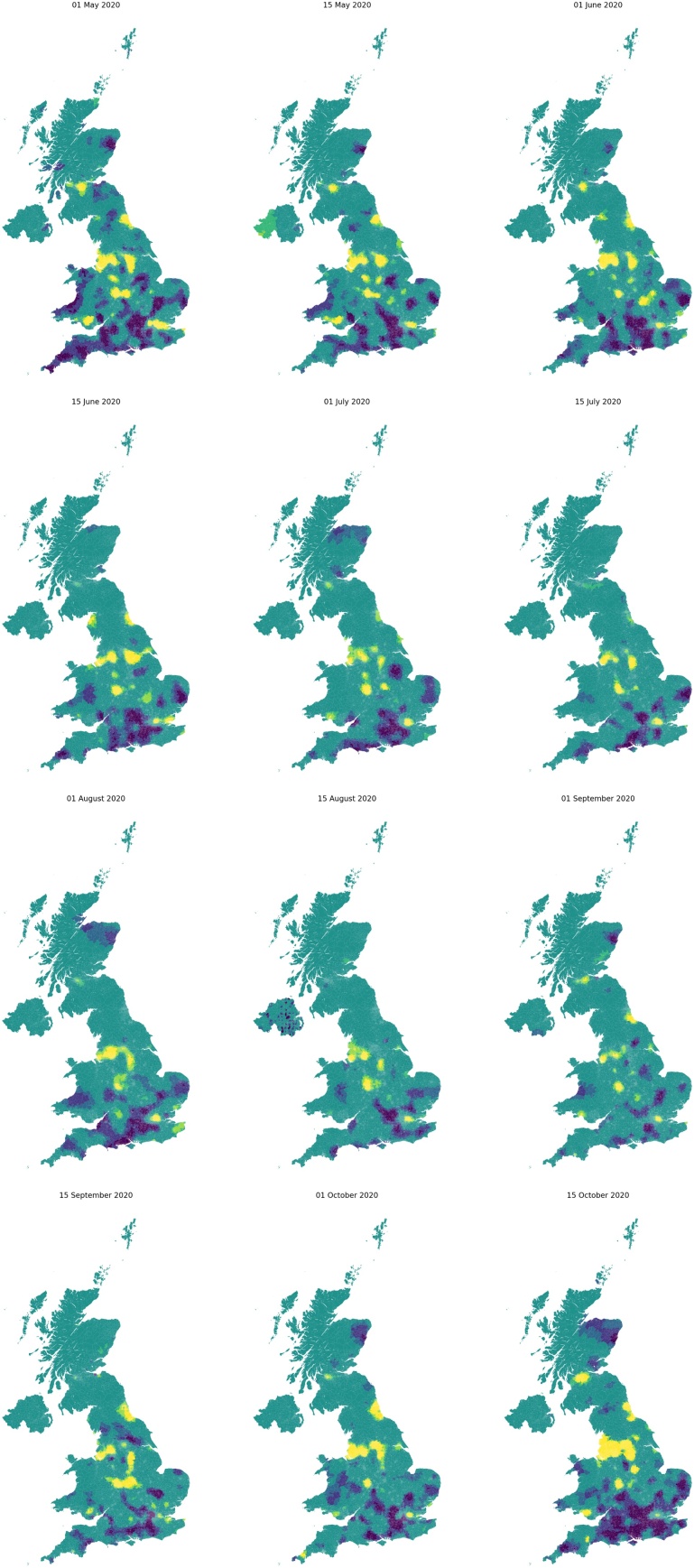
Predictive probabilities for LSOA-level prevalence to exceed the UK-wide average using a 14-day window 1st May – 1st November 2020. Purples represent low probabilities, green mid range and yellow high probabilities. (For interpretation of the references to color in this figure legend, the reader is referred to the web version of this article.)

Predictive mapping at LSOA-level geography based on inputs derived using the prevalence algorithm described in Menni et al.[15] revealed small-scale spatial variation in disease prevalence, which varied over time. Figures 3 and 4 show UK-wide LSOA-level maps of predicted prevalence and predictive probability that each LSOA exceeded the national average prevalence, over the pandemic to 1st November 2020. Most hot-spots (bright yellow areas in Fig. 4) were located in or close to major cities, with Aberdeen and Bristol as notable exceptions.

**Fig. 5:**
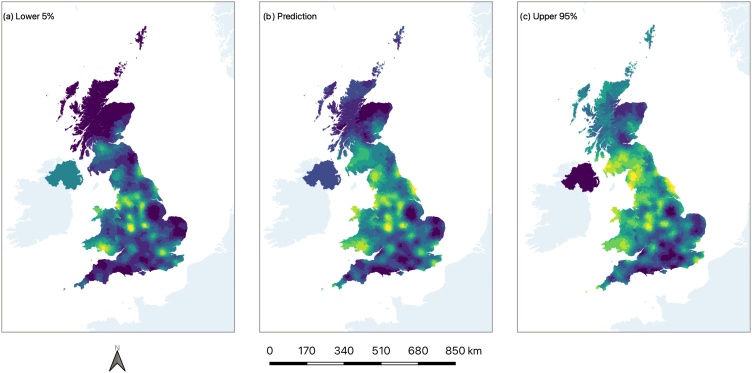
Predicted (lower 5% (left-hand panel (a)), mean (centre-panel (b)), and upper 95% (right-hand panel (c))) limits for the LSOA-level prevalence over the 14-day period ending 1st July 2020. Purples represent low values, green mid range and yellow high values. Maps coloured using Jenks Natural Breaks algorithm to highlight differences. (For interpretation of the references to color in this figure legend, the reader is referred to the web version of this article.)

Prevalence and exceedance probability maps need to be interpreted in combination. The prevalence exceedance (hot-spot) maps focus attention on areas that show statistically significant deviations above the national averages. However, all other things being equal, these were likely to occur in areas that have high population density and, consequently, deliver more precise local predictions. The three-panel format of Fig. 5 facilitates this combined interpretation by allowing the reader to check whether areas indicated in Fig. 4 as hot-spots are also areas whose predicted prevalence is markedly high. The left-hand (5% quantile) and right-hand (95% quantile) panels accompanying the predicted prevalence panel act as a guard against over-interpretation of imprecise point predictions. For example, the centre-panel of Fig. 5 shows that the largely rural area of west Cumbria had relatively high prevalence over the 14-day period ending 1st July, but the associated probability limits were wide, and the corresponding date in Fig. 4 does not indicate any part of west Cumbria to have been a hot-spot. Conversely, over this same period, prevalence levels in London were no longer among the highest in the country (Fig. 5, centre panel), but were nevertheless almost certainly above the English national average (Fig. 4) and therefore a hotspot.

## 4 Discussion

The COVID-19 pandemic has shown how a combination of skills (Health Informatics, Statistics and Geography) can provide insights to inform local and national government policy at a UK level. Building on the HDR UK ’One Institute’ principles, we have prototyped and delivered data infrastructures and analysis pipelines capable of delivering timely and insightful analytics to all levels of government. These have formed a cornerstone of the COVID-19 response in Scotland and Wales in particular, and illustrate how fine-grained spatio-temporal inferential mapping tools for near-real-time crowd-sourced georeferenced health outcome data are critical to inform rapid public health responses.

The platform established within the SAIL system should not be seen as uniquely applicable to data from the COVID Symptom Study app, or indeed to COVID-19. Such tools can be applied to any source of georeferenced health data, including when comparing data sourced via different detection and testing platforms, each of which may have a different target population, sensitivity and specificity. We note the particular relevance to newly emerging infectious conditions – where spatiotemporal mapping of spread is critical – and thus the need to maintain such platforms so that they can be activated as each pandemic arises, e.g. for influenza [19]. The required geospatial methodology for these tools has been available since the early years of the century [20]. Diggle et al. [21] report on a real-time surveillance system for calls to the now-defunct NHS Direct on-line triage service [22] for which the primary reason for the call was recorded as non-specific gastro-intestinal illness. This system was developed in collaboration with the Southampton Public Health Service and ran in prototype form over the years 2001 to 2003.

There has been a proliferation of mapped outputs related to the COVID-19 pandemic with policy makers and the general public equally concerned with tracking the pandemic. However, most of the published maps have been at a regional level [7], [5], [23]. Although valuable, these can mask localised hotspots as exemplified by the Leicester, UK outbreak. In situations where expedient decisions are required, mapping statistical outputs to granular geographies, with supporting information relating to confidence intervals, gives policy makers better information to take necessary action. Also, by recognising and exploiting spatial correlation in the underlying prevalence surface, geospatial statistical methods can deliver substantially more precise estimation of local prevalence than classical methods that implicitly assume independence of outcomes in different spatial units [24].

There is an extensive literature on statistical models for geo-referenced prevalence data. All exploit the fact that in the presence of spatial correlation, prevalence data from any location are partially predictive of prevalence at nearby locations. The most important way in which they differ is the spatial scale on which they operate. Spatially discrete, Markov random field models [25], [26] are widely used to construct disease atlases from data recorded as case-counts and denominators from a set of administrative regions that partition the area of interest. They typically define the spatial dependence amongst regions by their contiguities. In contrast, geostatistical models [10] and point process models [27], [28] are spatially continuous and define spatial dependence between locations as a function of their distance apart. Geostatistical models treat the data as case-counts and denominators from a set of sampling locations within the area of interest, whereas point process models require individual cases to be accurately georeferenced; for example, by full UK post-code. In our case, we could have used individual case data but preferred to aggregate to LSOA-level to acknowledge that, even in lockdown, most members of the population were not rigidly confined to their homes. However, we did not want to use contiguities to determine spatial dependence because of the very wide variation in the geographical sizes of LSOAs in different parts of the UK. For these reasons, we analysed the data using a geostatistical model with population-weighted LSOA centroids as the nominal sampling locations.

The use of predictive probability mapping is an important feature of our approach as it enables the production of maps that relate directly to public health policy. This concept is well-established in control programmes for a number of neglected tropical diseases; for example, WHO guidelines [29] for prophylactic treatment of soil-transmitted helminth infections specify different levels of continued treatment according to the exceedance or not of a set of agree prevalence thresholds. At the time of writing, the UK uses a “tiered” system of Covid risk-levels to determine what restrictions are in place in each part of the country, but the criteria for allocating tier membership are opaque with large administrative boundaries used to define which communities are included.

We acknowledge the limitations that come with using self-reported symptom data from an app used voluntarily. Firstly, confirmation that self-reported symptoms did indeed represent COVID-19 disease was not possible at a UK level, although the multi-symptom algorithm utilised was generated using predictive regression modelling comparing symptoms to self-reported reverse transcription polymerase chain reaction SARS-CoV-2 test results [15]. Secondly, the individuals included in the studied population are not a random sample of the UK population, potentially presenting a source of collider bias due to the link between age and app usage [30], nor are they necessarily representative with respect to other factors that are either known, or thought likely, to affect susceptibility; for example, gender or ethnicity. The inclusion in the model of LSOA-level covariate information is a potential route to controlling for these at LSOA level, although not at individual level. For example, in the current COVID-19 context information on the age distribution of app users would allow adjustment for the potentially non-representative sub-population of active app users. For environmentally driven health outcomes, such as asthma symptom exacerbation in relation to air quality, covariate adjustment could also materially improve predictive precision. In this paper we use repeated cross-sectional analysis of the app data to visualise change in prevalence across the UK. However, other approaches which borrow information over time as well as space [31], [32], could be considered when measuring longitudinal change in predicted prevalence.

This combination of real-time data sources and rapid analytical tools using readily adaptable methodologies is a powerful one in the control of pathogens where evolving spatio-temporal patterns of incidence are of public health concern. Our response to COVID-19 has much to teach us about preparedness for the next pandemic [19], [33].

## 5 Conclusions

In conclusion, we have demonstrated the value of a real-time spatio-temporal inferential mapping platform for public health efforts during the emergence and spread of infectious diseases. The work has been conducted in the confines of the privacy-protecting SAIL Databank to produce statistically robust results at a spatially granular level whilst ensuring that no individual contributor to the ZOE Symptom Study app can be identified. Such tools are not only essential to produce population-weighed estimates of disease prevalence, but they provide a unique insight into the geographical distribution of the disease, thus informing local and national control efforts.

## Summary points

**What is known?**

• COVID-19 has highlighted the need for robust methods for identifying outbreaks of disease and local levels.
• Most mapping efforts have so far been restricted to regional level estimates – there are very few local level estimates of COVID-19 prevalence.
• Self reported app data is currently being contributed by 4 million people in the UK.

**What we are adding?**

• We demonstrate the use of sophisticated spatial modelling for near-real-time prediction of COVID-19 prevalence at small-area resolution to inform strategic government policy areas.
• We provide estimates of their precision, to guard against over-reaction to potentially spurio us features of /’best guess/’ predictions.
• We demonstrate that adapting existing geospatial statistical methods, originally developed for global health applications, can be used in an anonymised databank environment, thus preserving the privacy of the individuals who contribute their data.

## Conflicts of interest

The authors declare no conflicts of interest.
