# Supplementary material for: Real-time spatial health surveillance: Mapping the UK COVID-19 epidemic
Source: Int J Med Inform. 2021 May;149:104400. doi: 10.1016/j.ijmedinf.2021.104400 (PMC7843148; doi:10.1016/j.ijmedinf.2021.104400)
Supplement: Supplementary file 1 [file mmc1.pdf]

Supplementary material: Geospatial statistical model and inference

S.1 Model formulation

The standard way to relate prevalence – the probability of a positive outcome – to one or more variable risk-factors is a logistic regression equation. For example, writing $P(x)$ for the probability of a positive outcome at a location x within a specified time-interval, if geographical variation in prevalence were thought to be primarily associated with deprivation, a candidate logistic regression model would be

$$\log[P(x)/\{1 - P(x)\}] = \alpha + \beta \times \text{deprivation score} \tag{1}$$

In a geospatial model, equation (1) is extended to

$$\log[P(x)/\{1 - P(x)\}] = \alpha + \beta \times \text{deprivation score} + S(x), \tag{2}$$

where now $S(x)$ is an unobserved, spatially correlated stochastic process. Its role is to account for that part of the geographical variation in prevalence that is *not* explained by available covariates. Its inclusion in the model has two potentially important consequences: it guards against invalid inferences on covariate effects by acknowledging that data from different locations may not be statistically independent; more importantly in the current context, it delivers more precise estimates of the underlying prevalence surface $P(x)$ by transferring some of what would otherwise have been treated as unpredictable random variation into predictable, spatially correlated variation. The extent to which these potential benefits are realised in practice is context-specific – the stronger the spatial correlation structure, the greater the benefits. This makes it essential that the spatial correlation structure is estimated from the data, rather than being pre-specified.

S.2 Exploratory analysis

In preliminary analyses of data from different parts of the UK we did not find a robust association of app-reported symptom prevalence with LSOA-level deprivation or population density. We therefore fitted a version of the geospatial model (2) without covariate-adjustment, i.e. all geographical variation in app-reported symptom prevalence is ascribed to the stochastic term $S(x)$.

To explore the spatial correlation structure of the data, we converted the positive symptom report count, y, and the number of active users, n, for each LSOA in a given 14-day time-window into empirical logits, $el = \log\{(y + 0.5)/(n - y + 0.5)\}$ and calculated their *variogram*, as follows. Associate each empirical logit, el_i, with the population-weighted centroid, x_i, of the corresponding LSOA. Calculate the quantities $v_{ij} = \frac{1}{2}(el_i - el_j)^2$ and $d_{ij} = ||x_i - x_j||$, the distance between x_i and x_j. Group the d_{ij} in a set of distance bins centred on $u_k : k = 1, ..., m$ and calculate $\bar{v}_k$ as the average of all of the v_{ij} whose corresponding d_{ij} fall within the kth distance bin. A plot of $\bar{v}_k$ against u_k, called the *empirical variogram*, estimates the quantity $V(u_k) = \tau^2 + \sigma^2\{1 - \rho(u)$, where σ^2 is the variance of $S(x)$, $\rho(u)$ is the correlation between pairs $S(x)$ and $S(x')$ at locations a distance u apart, and $\tau^2 > 0$ represents the sampling variation in the individual el_i. Figure 6 shows the result using square-root-transformed empirical logits from Scotland's data over the 14-day time-window 1 to 14 April 2020.

Based on Figure 6 and similar results from other parts of the UK we used a standard specification for $S(x)$ as a Gaussian process with twice-differentiable Matérn correlation function (Diggle and Giorgi, 2019, p32), giving a model with three parameters: the intercept, α, in (2), the variance, σ^2, of the stochastic process $S(x)$, which represents how variable are the log-odds of prevalence from place to place; and a correlation parameter, ϕ, which determines the rate at which the correlation between log-odds of prevalence at two locations decays as the distance between them increases.

S.3 Inference

We estimated the model parameters by Monte Carlo maximum likelihood, as implemented in

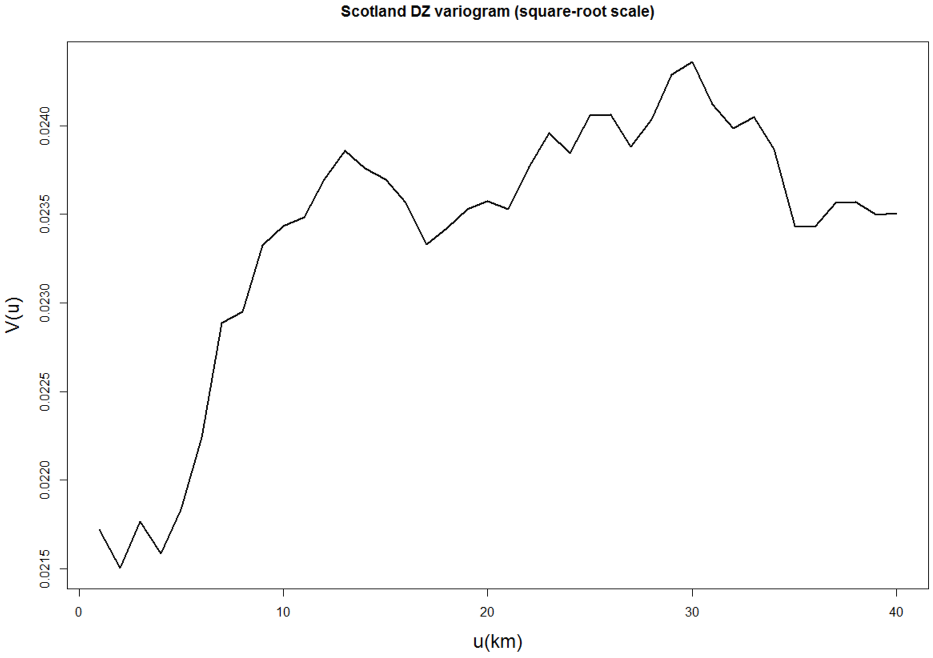

Figure 6: Empirical variogram for empirical logits of positive symptom reports in Scotland over the 14-day time-window 1 to 14 April 2020.

the `R` package `PrevMap` (Giorgi and Diggle, 2017). This method ensures statistical efficiency, but requires the tuning of a Markov chain Monte Carlo algorithm. We ran the chain with 11,000 samples, from which the first 1,000 were discarded and the remainder sampled every tenth iteration, leaving a sample of size 1,000 from the joint predictive distribution of the prevalences $P(x)$ for each LSOA. Initial values of the model parameters were taken from visual inspection of the empirical variogram shown in Figure 6, but optimised separately for each time-window of data in each of the UK's constituent countries.

Diggle, P.J. and Giorgi, E. (2019). *Model-based Geostatistics: Methods and Applications in Global Public Health*. Boca Raton: CRC Press

Giorgi, E. and Diggle, P.J. (2017). PrevMap: an R package for prevalence mapping. *Journal of Statistical Software*, **78**, 1-29, doi:10.18637/jss.v078.i08

Summary Table

What is known
• COVID-19 has highlighted the need for robust methods for identifying outbreaks of disease and local levels<br>• Most mapping efforts have so far been restricted to regional level estimates - there are very few local level estimates of COVID-19 prevalence<br>• Self reported app data is currently being contributed by 4 million people in the UK
**What we are adding**
• We demonstrate the use of sophisticated spatial modelling for near-real-time prediction of COVID-19 prevalence at small-area resolution to inform strategic government policy areas<br>• We provide estimates of their precision, to guard against over-reaction to potentially spurio us features of /'best guess/' predictions<br>• We demonstrate that adapting existing geospatial statistical methods, originally developed for global health applications, can be used in an anonymised databank environment, thus preserving the privacy of the individuals who contribute their data.
